# Supplementary material for: Design principles for accurate folding of DNA origami
Source: bioRxiv. 2024 Mar 19:2024.03.18.585609. Preprint. [Version 1] doi: 10.1101/2024.03.18.585609 (PMC10983894; doi:10.1101/2024.03.18.585609)
Supplement: Supplement 1 [file NIHPP2024.03.18.585609v1-supplement-1.pdf]

bioRxiv preprint doi: https://doi.org/10.1101/2024.03.18.585609; this version posted March 19, 2024. The copyright holder for this preprint (which was not certified by peer review) is the author/funder, who has granted bioRxiv a license to display the preprint in perpetuity. It is made available under aCC-BY 4.0 International license.

Supplementary Information for

Design principles for accurate folding of DNA origami

Tural Aksel[1]*, Erik J. Navarro[1], Nicholas Fong[1], Shawn M. Douglas[1]*
1 Department of Cellular and Molecular Pharmacology
University of California, San Francisco
* turalaksel@gmail.com and shawn.douglas@ucsf.edu

Contents

Figure S1 | Breakpoint Rules 2
Figure S2 | 10×10 block heatmaps 3
Figure S3 | 16×4 block heatmaps 4
Figure S4 | 8×8 block heatmaps 5
Figure S5 | 4×16 block heatmaps 6
Figure S6 | Detailed gel analysis 7
Figure S7 | 10×10 block TEM image 8
Figure S8 | 4×16 block TEM image 9
Figure S9 | 8×8 block TEM image 10
Figure S10 | 16×4 block TEM image 11
Figure S11 | Per-staple thermodynamic analysis 12
Figure S12 | Scaffold permutation score distributions 13

bioRxiv preprint doi: https://doi.org/10.1101/2024.03.18.585609; this version posted March 19, 2024. The copyright holder for this preprint (which was not certified by peer review) is the author/funder, who has granted bioRxiv a license to display the preprint in perpetuity. It is made available under aCC-BY 4.0 International license.

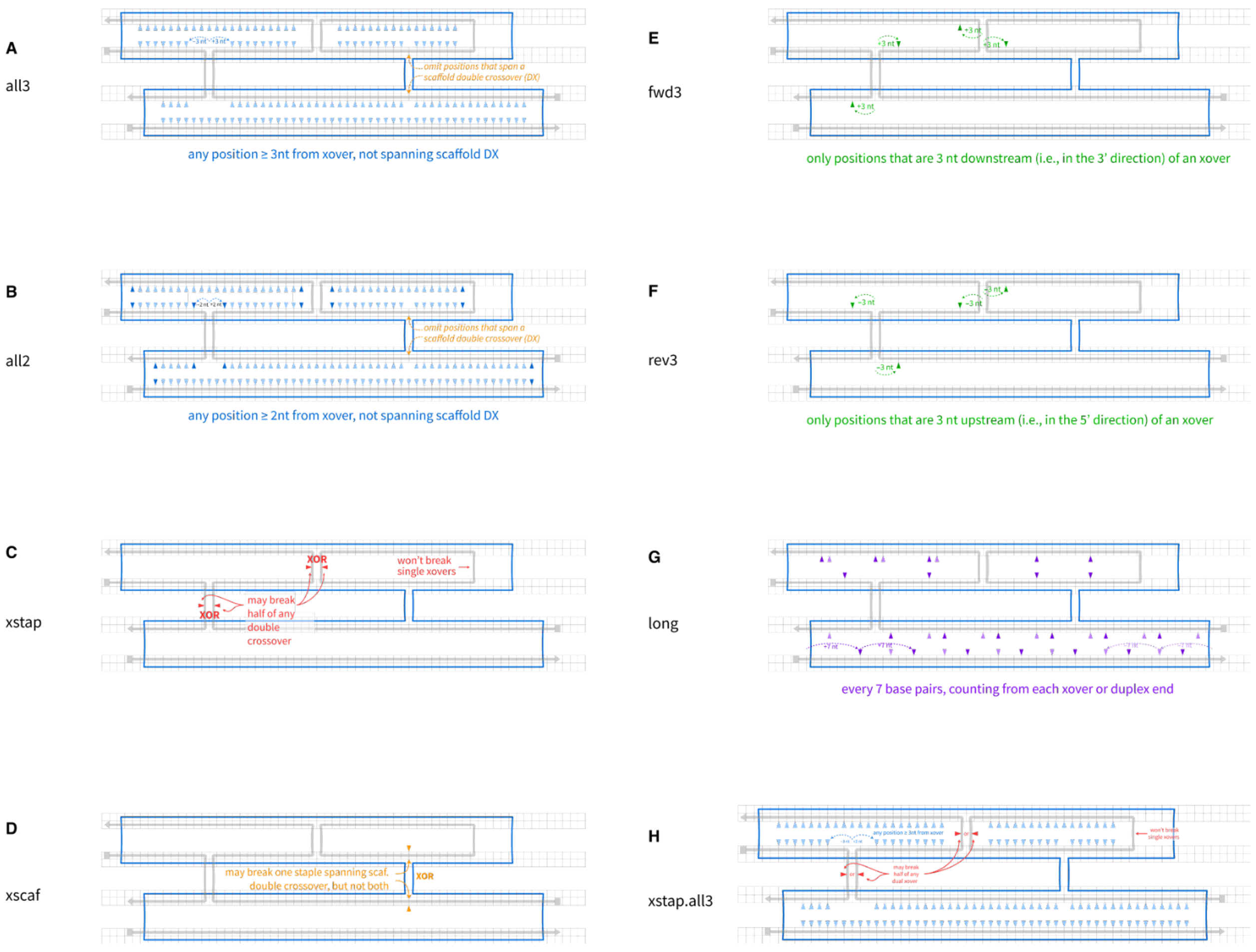

Figure S1 | Breakpoint Rules

To identify the design principles driving folding accuracy, we needed a deterministic approach to encode and compare design rules. We devised a rule-based system for specifying the allowed staple 'breakpoints,' or locations where a precursor staple route is broken between two nucleotides, leaving a pair of 5' and 3' endpoints. Breakpoint rules are used in the construction of the weighted graph (Fig. 1A). We examined the following rules: (**A**) Rule 'all3' includes any breakpoint position ≥ 3nt from a staple crossover (xover), and not spanning a scaffold double crossover (DX). (**B**) Rule 'all2' includes any breakpoint position ≥ 2nt from a staple xover, and not spanning a scaffold DX. (**C**) Rule 'xstap' allows for breaking half of any staple DX. (**D**) Rule 'xscaf' allows for breaking one of the staples spanning a scaffold DX, but not both. (**E**) Rule 'fwd3' includes only positions that are 3 nt downstream (i.e., in the 3' direction) of an xover. (**F**) Rule 'rev3' includes only positions that are 3 nt upstream (i.e., in the 5' direction) of an xover. (**G**) Rule 'long' includes a breakpoint every 7 nucleotides pairs, counting from each xover or duplex end. (**H**) Rule 'xstap.all3' is a composite rule that includes the union of all breakpoints allowed by the 'xstap' and 'all3' rules.

bioRxiv preprint doi: https://doi.org/10.1101/2024.03.18.585609; this version posted March 19, 2024. The copyright holder for this preprint (which was not certified by peer review) is the author/funder, who has granted bioRxiv a license to display the preprint in perpetuity. It is made available under aCC-BY 4.0 International license.

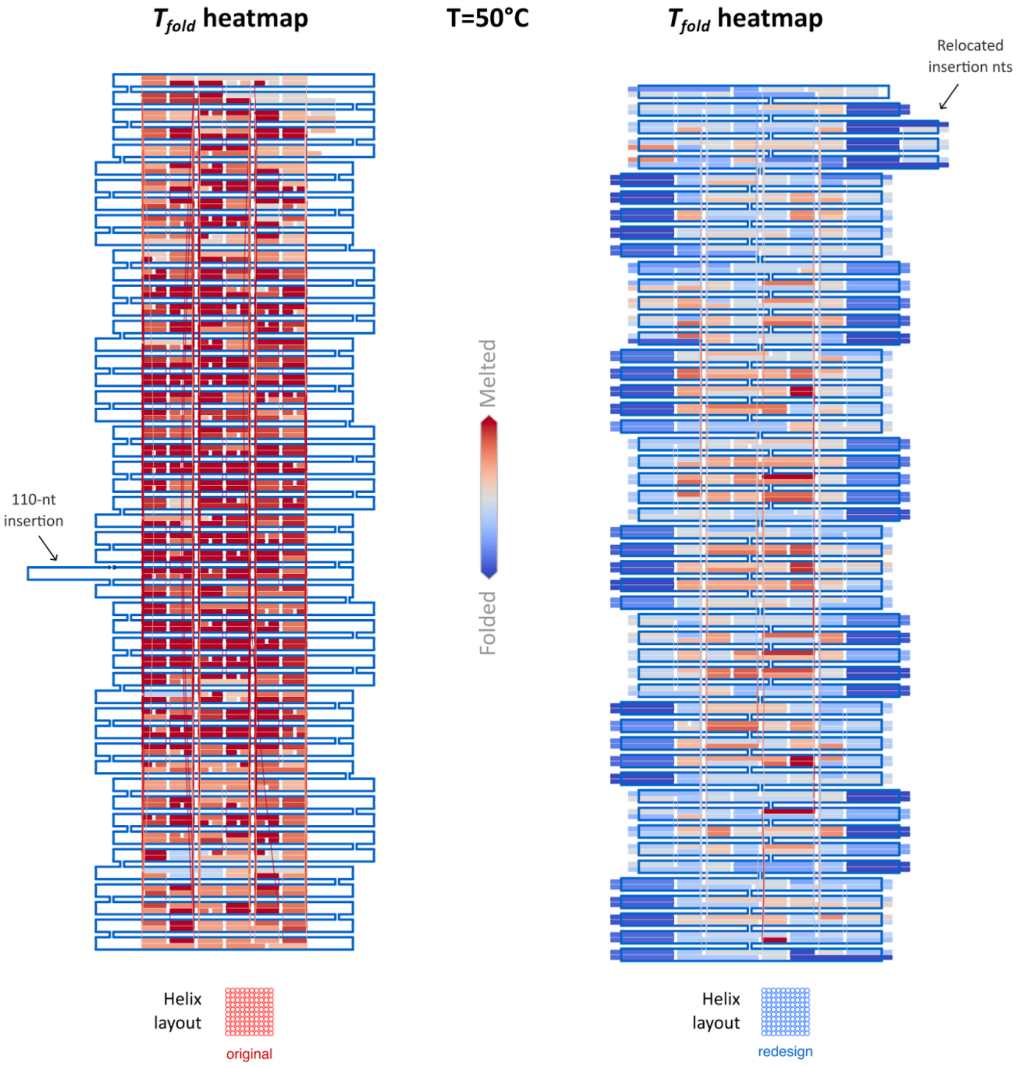

Figure S2 | 10×10 block heatmaps

To mitigate aggregation, the original design relied on a passivation strategy of leaving unpaired scaffold nucleotides at the left and right edges. A total of 2725 scaffold nucleotides are left unpaired, including a 110-nt 'insertion' at the helix[base] position 56[30]; the remaining 4835 scaffold bases form duplexes with staple strands.

In our redesign, we rerouted the internal scaffold crossovers to a 'middle-seam' layout, and relocated the 110-nt insertion domain to extend helices 4, 5, 6, 7, 8, and 9 on the right edge. Before applying our staple-routing algorithm, we added edge staples that utilize unpaired 'TTT' overhangs for aggregation, resulting in 2719 added base pairs (6 of 7560 remain unpaired). The added edge staples in the redesigned 10×10 block have low ΔG_{loop} penalties due to the revised scaffold crossover layout, resulting in the overall decrease of the median ΔG_{loop} scores shown in Fig. 4B.

bioRxiv preprint doi: https://doi.org/10.1101/2024.03.18.585609; this version posted March 19, 2024. The copyright holder for this preprint (which was not certified by peer review) is the author/funder, who has granted bioRxiv a license to display the preprint in perpetuity. It is made available under aCC-BY 4.0 International license.

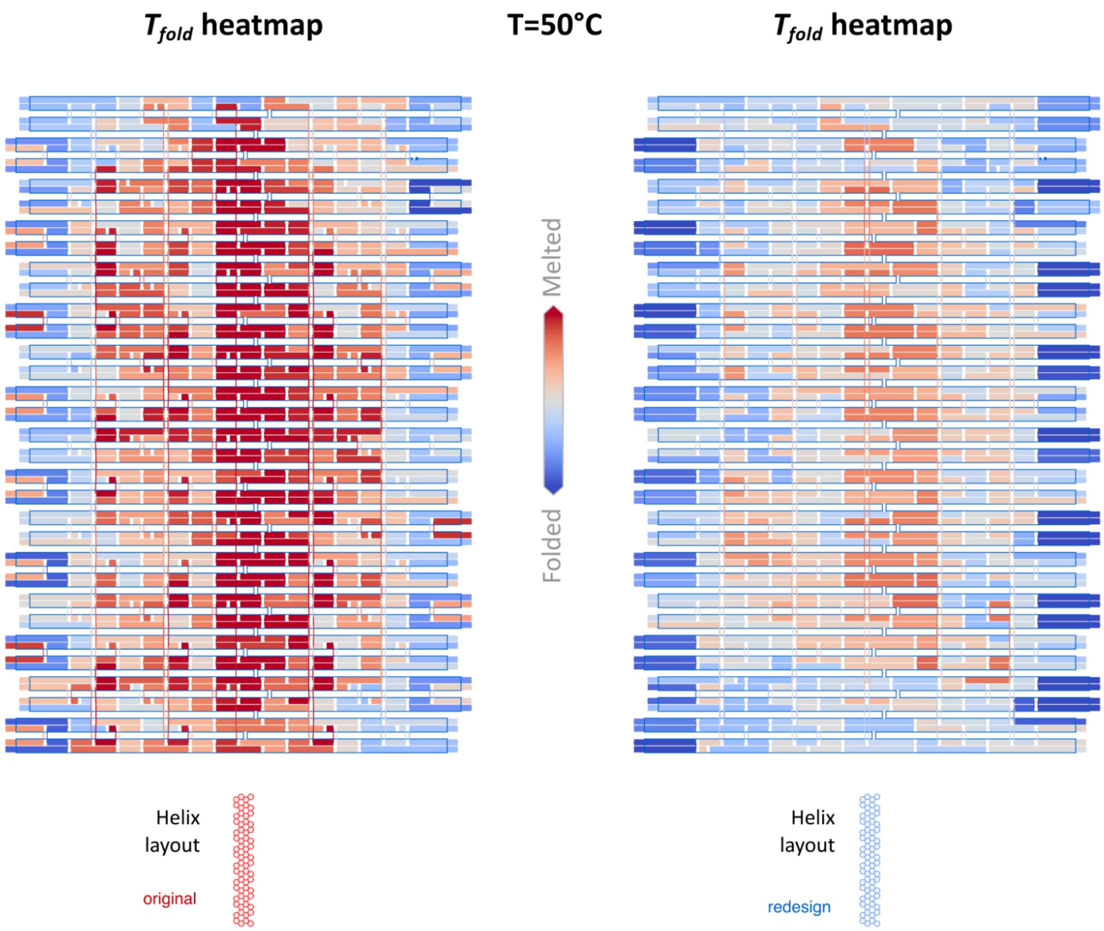

Figure S3 | 16×4 block heatmaps

The 16×4 blocks used identical scaffold routes and staple-passivation strategies.

bioRxiv preprint doi: https://doi.org/10.1101/2024.03.18.585609; this version posted March 19, 2024. The copyright holder for this preprint (which was not certified by peer review) is the author/funder, who has granted bioRxiv a license to display the preprint in perpetuity. It is made available under aCC-BY 4.0 International license.

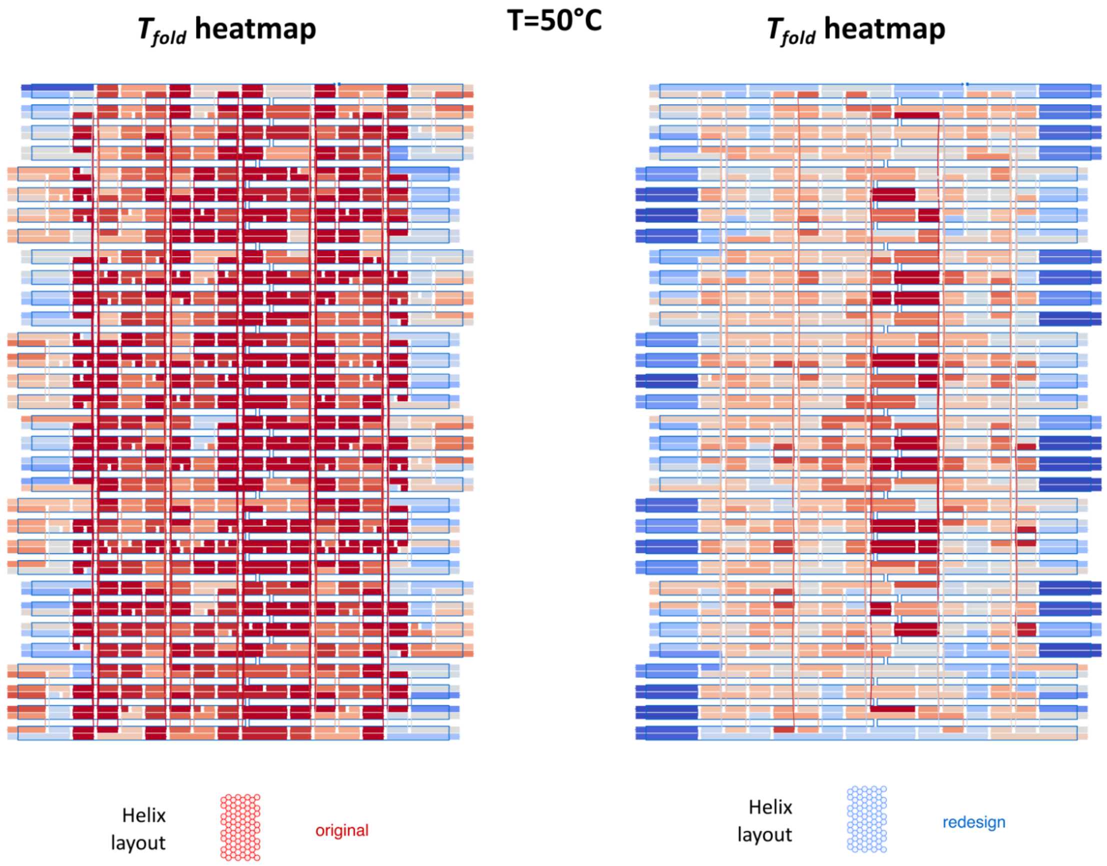

Figure S4 | 8×8 block heatmaps

The 8×8 blocks used identical scaffold routes and staple-passivation strategies.

bioRxiv preprint doi: https://doi.org/10.1101/2024.03.18.585609; this version posted March 19, 2024. The copyright holder for this preprint (which was not certified by peer review) is the author/funder, who has granted bioRxiv a license to display the preprint in perpetuity. It is made available under aCC-BY 4.0 International license.

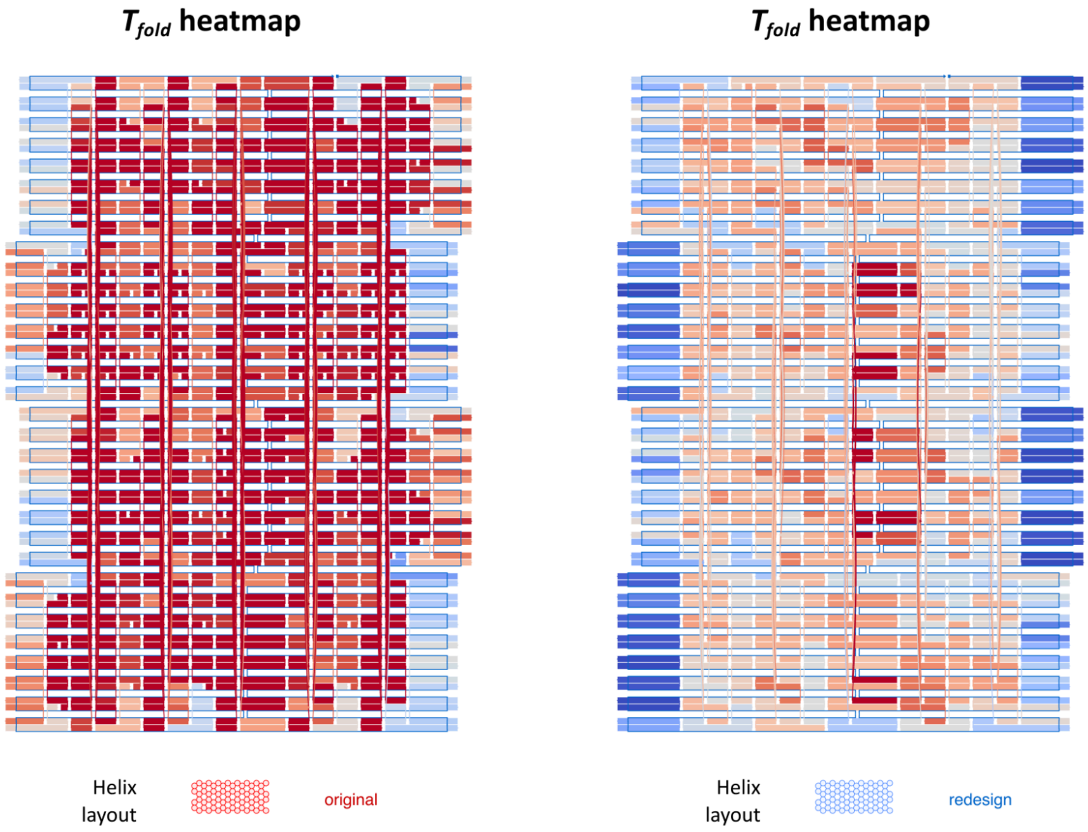

Figure S5 | 4×16 block heatmaps

The 4×16 blocks used identical scaffold routes and staple-passivation strategies.

bioRxiv preprint doi: https://doi.org/10.1101/2024.03.18.585609; this version posted March 19, 2024. The copyright holder for this preprint (which was not certified by peer review) is the author/funder, who has granted bioRxiv a license to display the preprint in perpetuity. It is made available under aCC-BY 4.0 International license.

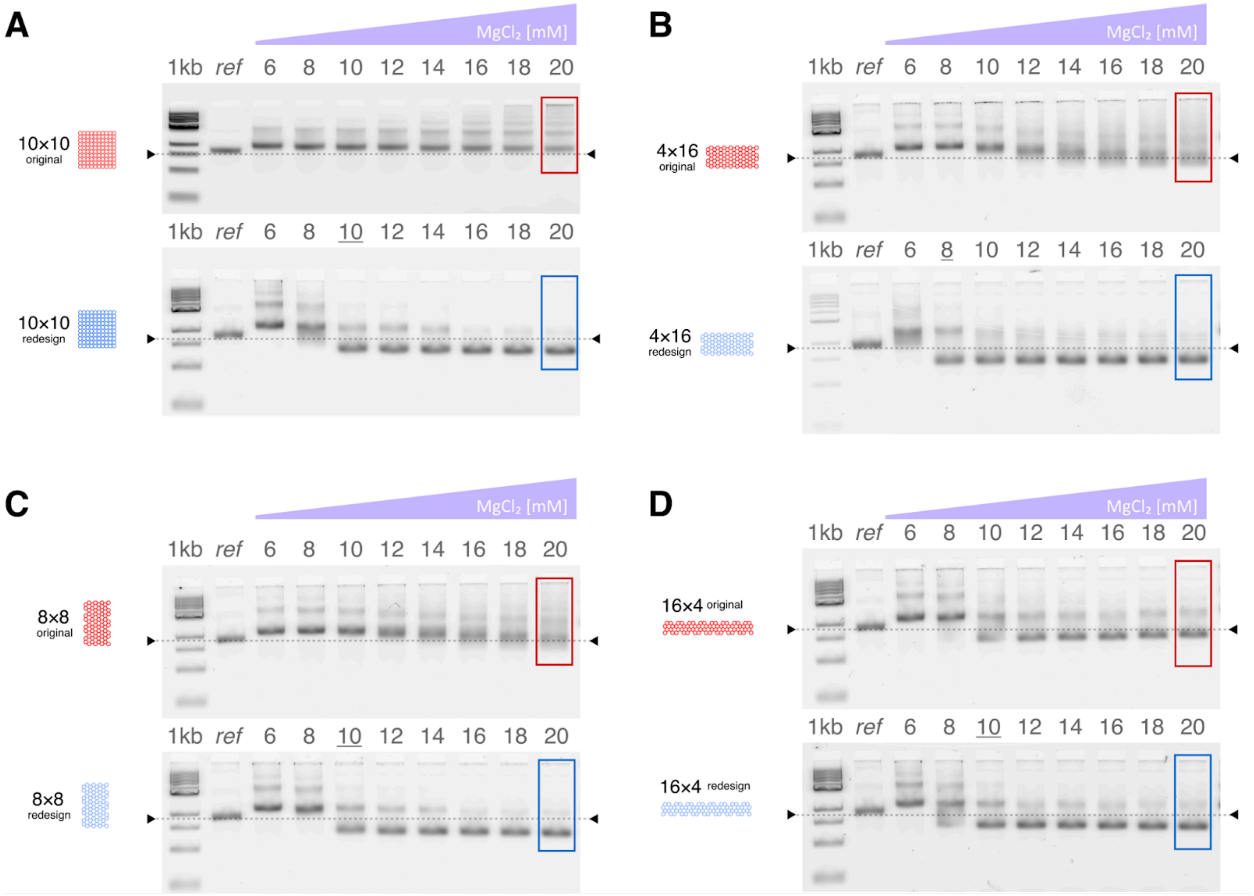

Figure S6 | Detailed gel analysis

(**A**) 10×10 block. (**B**) 4×16 block. (**C**) 8×8 block. (**D**) 16×4 block. We folded each design and ran on separate gels as described in the Materials and Methods section. Lane 2 (“ref”) in each gel contained p7650 (A) or p8064 (B–D) scaffold ssDNA as a reference band for alignment and scaling during image analysis. Lanes 3–10 are labeled according to $MgCl_2$ concentration. Intensity histograms of the boxed regions of the 20 mM $MgCl_2$ lanes are shown in Fig. 3.

bioRxiv preprint doi: https://doi.org/10.1101/2024.03.18.585609; this version posted March 19, 2024. The copyright holder for this preprint (which was not certified by peer review) is the author/funder, who has granted bioRxiv a license to display the preprint in perpetuity. It is made available under aCC-BY 4.0 International license.

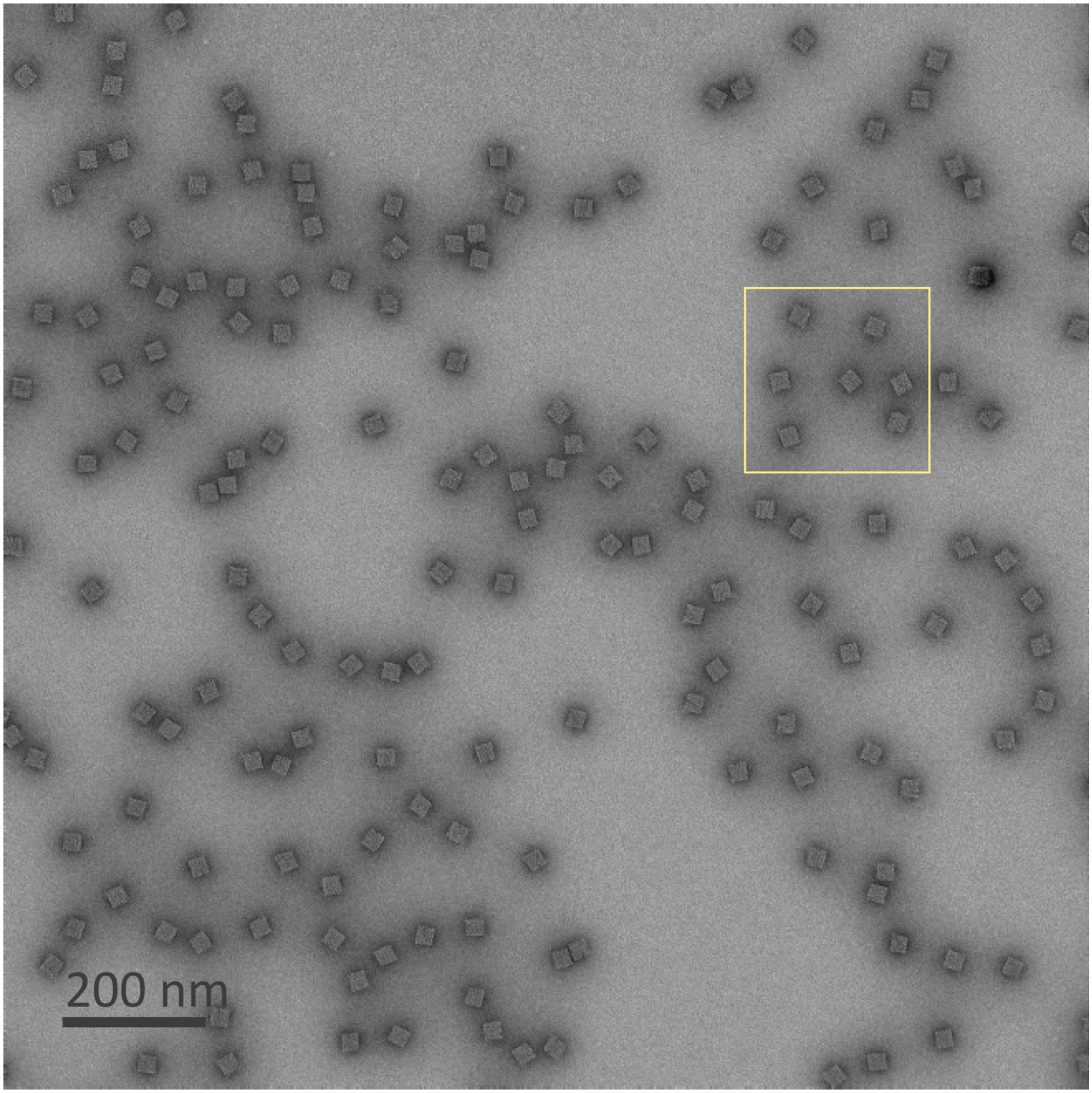

Figure S7 | 10×10 block TEM image

Negative stain. Boxed region is identical to Fig. 3A. Scale bar: 200 nm.

bioRxiv preprint doi: https://doi.org/10.1101/2024.03.18.585609; this version posted March 19, 2024. The copyright holder for this preprint (which was not certified by peer review) is the author/funder, who has granted bioRxiv a license to display the preprint in perpetuity. It is made available under aCC-BY 4.0 International license.

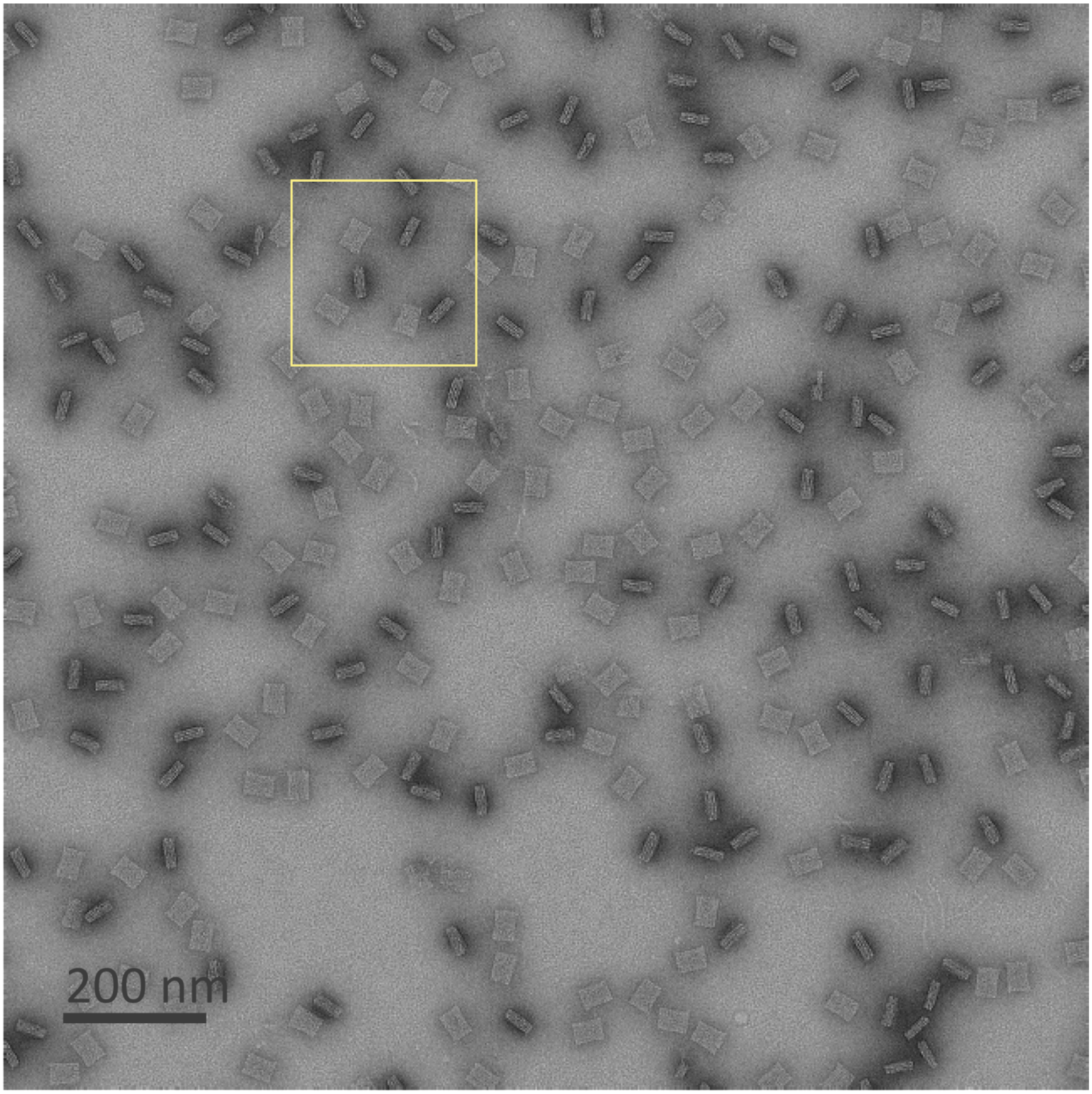

Figure S8 | 4×16 block TEM image

Negative stain. Boxed region is identical to Fig. 3B. Scale bar: 200 nm.

bioRxiv preprint doi: https://doi.org/10.1101/2024.03.18.585609; this version posted March 19, 2024. The copyright holder for this preprint (which was not certified by peer review) is the author/funder, who has granted bioRxiv a license to display the preprint in perpetuity. It is made available under aCC-BY 4.0 International license.

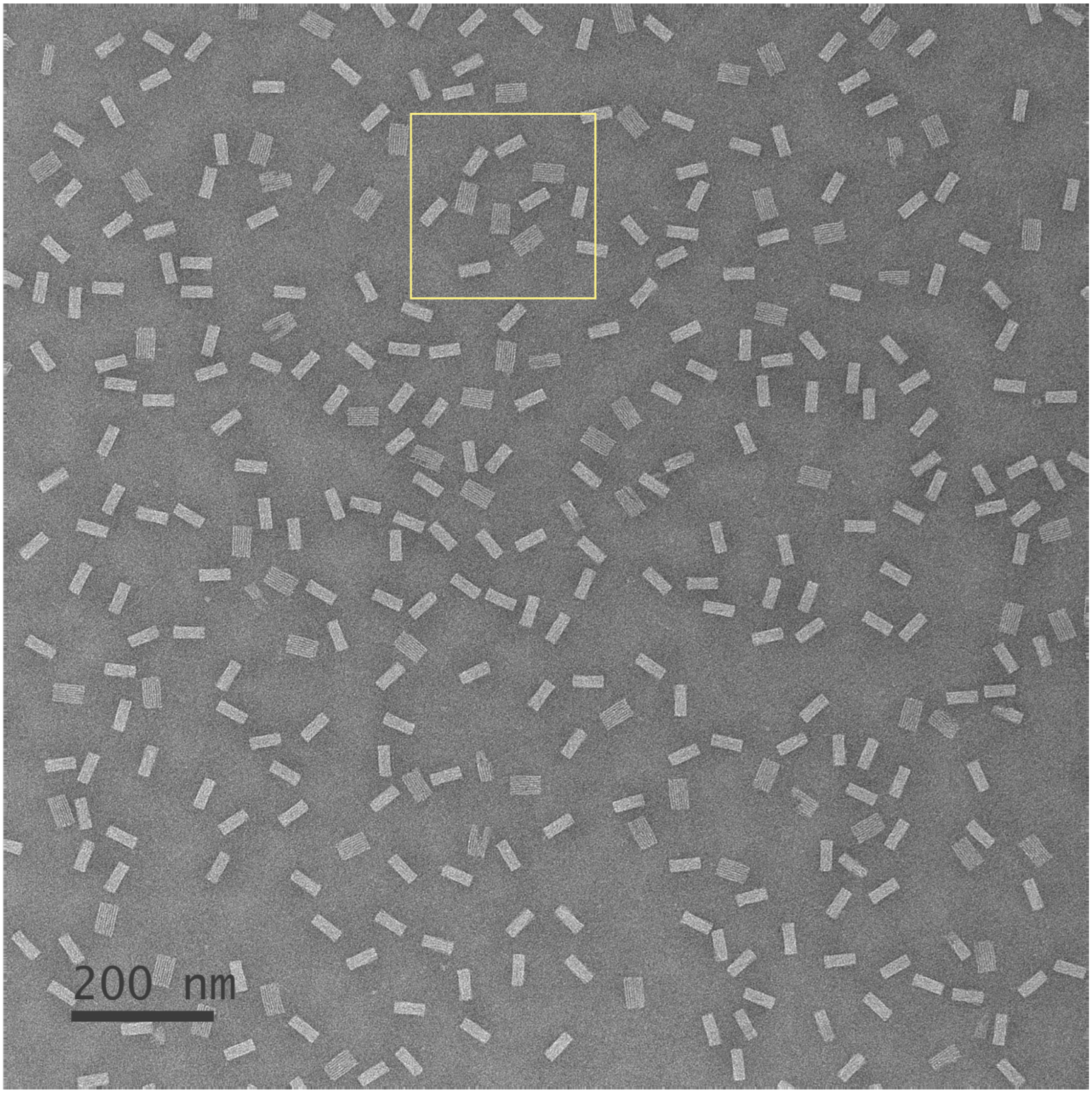

Figure S9 | 8×8 block TEM image

Negative stain. Boxed region is identical to Fig. 3C. Scale bar: 200 nm.

bioRxiv preprint doi: https://doi.org/10.1101/2024.03.18.585609; this version posted March 19, 2024. The copyright holder for this preprint (which was not certified by peer review) is the author/funder, who has granted bioRxiv a license to display the preprint in perpetuity. It is made available under aCC-BY 4.0 International license.

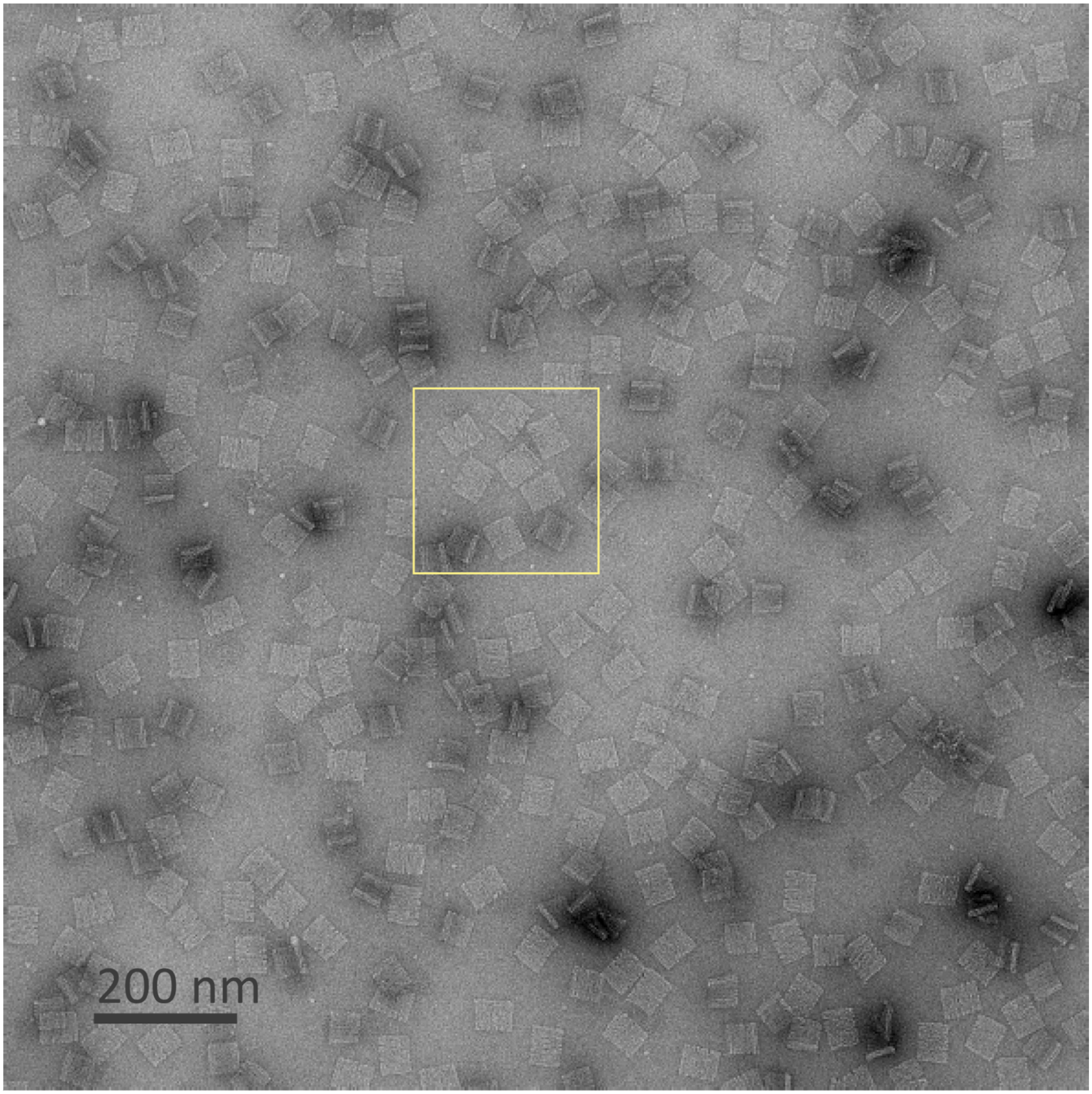

Figure S10 | 16×4 block TEM image

Negative stain. Boxed region is identical to Fig. 3D. Scale bar: 200 nm.

bioRxiv preprint doi: https://doi.org/10.1101/2024.03.18.585609; this version posted March 19, 2024. The copyright holder for this preprint (which was not certified by peer review) is the author/funder, who has granted bioRxiv a license to display the preprint in perpetuity. It is made available under aCC-BY 4.0 International license.

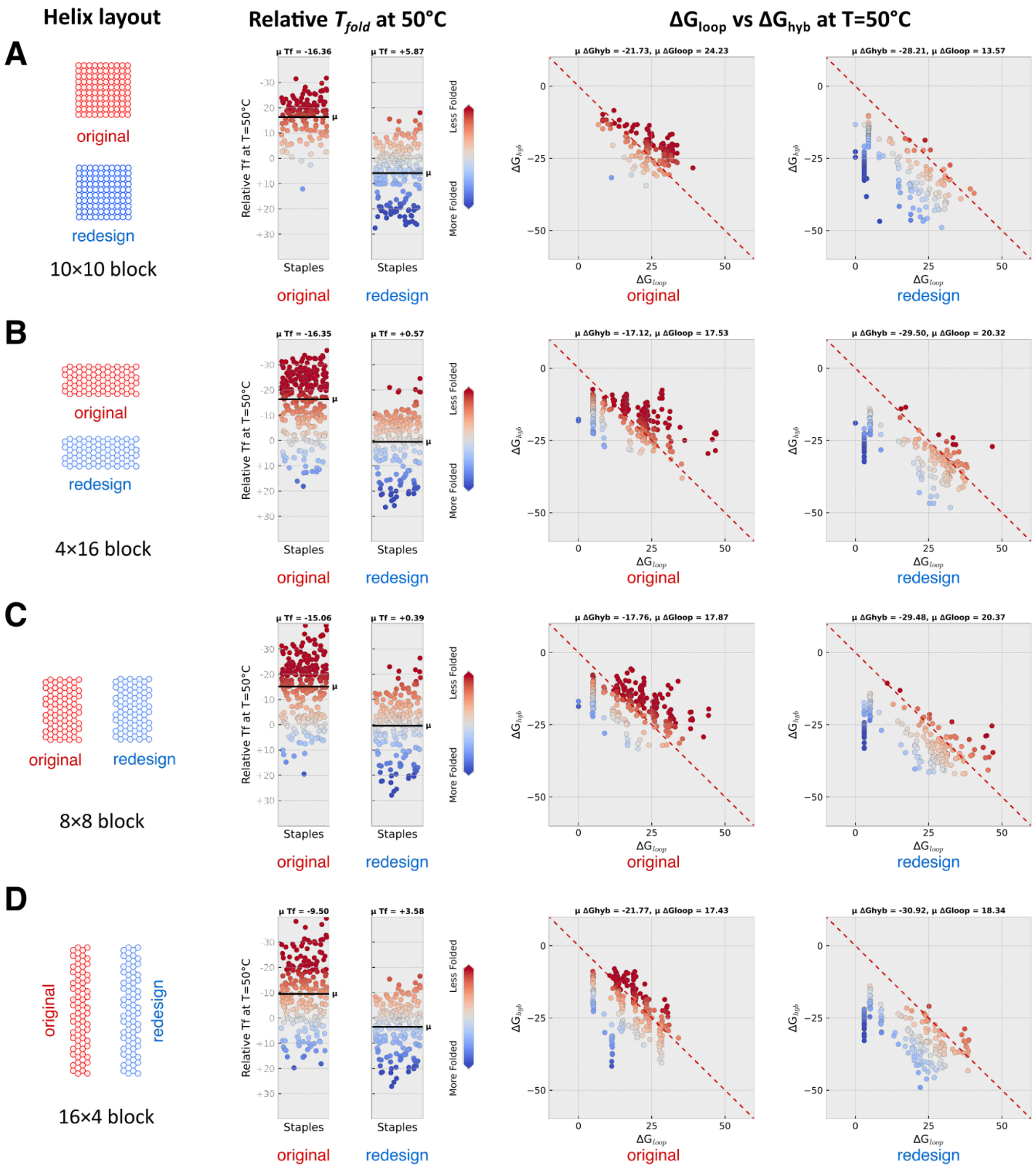

Figure S11 | Per-staple thermodynamic analysis

(**A**) 10×10 block. (**B**) 4×16 block. (**C**) 8×8 block. (**D**) 16×4 block. Strip plots show relative T_{fold} values at T=50°C. Color map ranges from +20°C (blue, more folded) to 0° (gray, 50% folded) to -20°C (red, less folded). Out-of-range values (>20°C or <-20°C) use the color of the closest in-range value. Mean (μ) T_{fold} values are indicated by a horizontal black line. Scatter plots show per-staple ΔG_{loop} (x-axis) and ΔG_{hyb} (y-axis), with points colored according to their T_{fold} values from the strip plots. Dashed line (red) indicates where $\Delta G_{loop} + \Delta G_{hyb} = 0$. Staples with values of $\Delta G_{loop} + \Delta G_{hyb} < 0$ appear below the diagonal and are predicted to fold at higher temperatures (blue points). The mid-range T_{fold} points (gray) appear slightly off the diagonal due to the ΔG_{bind} penalty term.

bioRxiv preprint doi: https://doi.org/10.1101/2024.03.18.585609; this version posted March 19, 2024. The copyright holder for this preprint (which was not certified by peer review) is the author/funder, who has granted bioRxiv a license to display the preprint in perpetuity. It is made available under aCC-BY 4.0 International license.

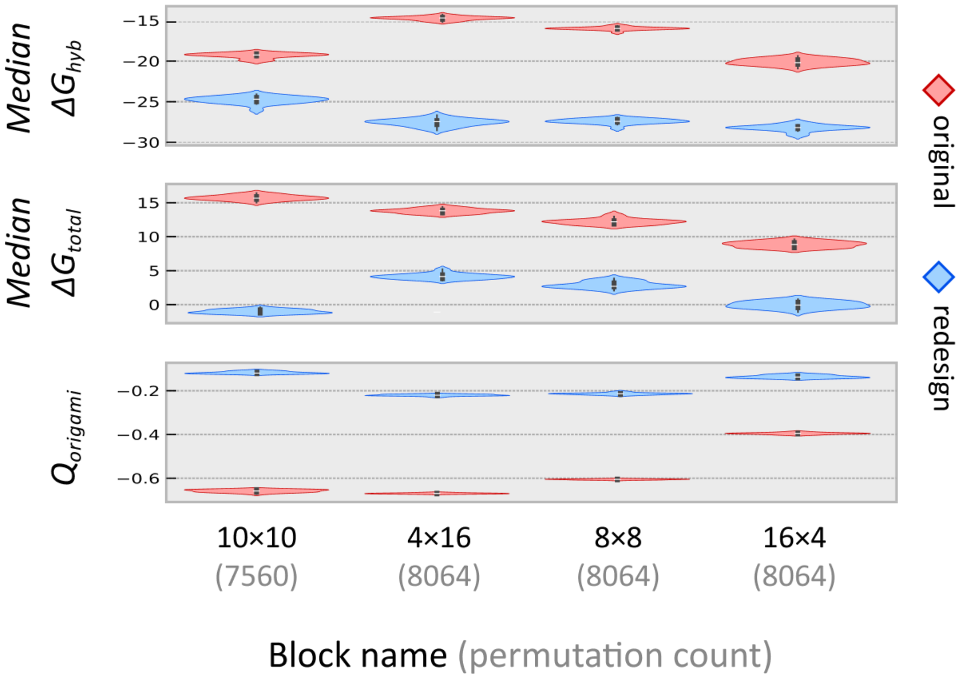

Figure S12 | Scaffold permutation score distributions

We calculated ΔG_{hyb} and ΔG_{total} scores for each design variant across all circular scaffold permutations. The *nth* scaffold permutation is generated by removing the first *n-1* nucleotides of the sequence and appending them to the end of the sequence. The global $Q_{origami}$ values are reproduced from Fig. 4C for comparison.
